# Seasonality and growth in tropical freshwater ectotherm vertebrates: Results from 1‐year experimentation in the African gray bichir, giraffe catfish, and the West African mud turtle

**DOI:** 10.1002/ece3.9936

**Published:** 2023-03-28

**Authors:** Axelle Gardin, Olga Otero, Elodie Réveillac, Alexandra Lafitte, Xavier Valentin, Florian Lapalus, Didier Bouchon, Géraldine Garcia

**Affiliations:** ^1^ PALEVOPRIM – UMR CNRS 7262 Université de Poitiers Bât. B35 – TSA 51106 6 rue Michel Brunet F‐86073 Poitiers Cedex 9 France; ^2^ LIENSs ‐ UMR CNRS 7266 La Rochelle Université ILE, 2 rue Olympe de Gouges 17000 La Rochelle France; ^3^ EBI – UMR CNRS 7267 Université de Poitiers TSA 51106 5 rue Albert Turpain F‐86073 Poitiers Cedex 9 France

**Keywords:** ectotherm vertebrates, environmental factors, seasonality, somatic growth, specific growth rate

## Abstract

Growth in ectotherm vertebrates is strongly rhythmed by seasonal variation in environmental parameters. To track the seasonal variation in ancient times in a continental and tropical context, we aim to develop a method based on the use of the growth rate of fossil ectotherm vertebrates (actinopterygians and chelonians) influenced by seasonal environmental fluctuations they experienced in their lifetime. However, the impact of environmental parameters on growth, positive or negative, and its intensity, depends on the taxa considered, and data are scarce for tropical species. For 1 year, an experiment was conducted to better understand the effect of seasonal variation in environmental parameters (food abundance, temperature, and photoperiod) on the somatic growth rate of three species of tropical freshwater ectotherm vertebrates: the fishes *Polypterus senegalus* and *Auchenoglanis occidentalis* and the turtle *Pelusios castaneus*. Mimicking seasonal shifts expected to be experienced by the animals in the wild, the experiment highlighted the preponderant effect of food abundance on the growth rate of those three species. Water temperature variation had a significant effect on the growth rate of *Po. senegalus* and *Pe. castaneus*. Moreover, the photoperiod demonstrated no significant effect on the growth of the three species. The duration of application of starvation or cool water conditions, ranging from 1 to 3 months, did not affect the growth rate of the animals. However, *Pelusios castaneus* showed a temporary sensitivity to the return of ad libitum feeding or of warm water, after a period of starvation or cool water, by a period of compensatory growth. Finally, this experiment revealed, in the three species, fluctuations in the growth rate under controlled and constant conditions. This variation, similar to the variation in precipitation and temperature observed in their native environment, could be linked to a strong effect of an internal rhythm controlling somatic growth rate.

## Graphical abstract

For 1 year, individuals belonging to three species of tropical ectotherm vertebrates (two fish species and one turtle species) were reared under controlled conditions. During this year, the food abundance, the temperature, and the photoperiod were modified independently to mimick seasonal variations and to identify their impact on the somatic growth rate of these animals. The food abundance and the temperature were generally positively correlated with growth, while the photoperiod did not impact the growth rate for those animals. Also, the experiment revealed a strong effect of an internal rhythm controlling somatic growth rate.

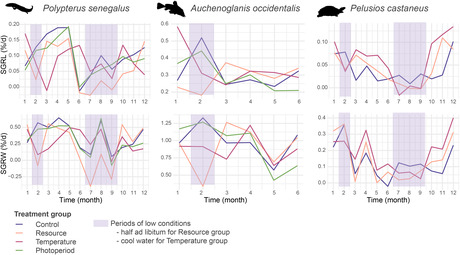

## 1 INTRODUCTION

Seasonal fluctuations of environmental parameters can affect many physiological functions, such as growth, in ectotherm vertebrates. The relationship between ectotherm's growth and isolated biotic and abiotic factors has been largely studied in multiple fields, for instance, developmental biology (Green & Fisher, 2004), ecology (e.g., Beckman et al., 1998; Rosenfeld et al., 2005), development of animal resources (Bœuf & Payan, 2001; Deane & Woo, 2009; Norberg et al., 2001), and paleontology or archaeology, to interpret the growth of animals as a paleoenvironmental proxy (Lapalus et al., 2018). Fossil growth patterns can aid in the evaluation of paleoenvironmental seasonal fluctuations, which are an important component of climate effects on past continental ecosystems (e.g., Tonkin et al., 2017; White & Hastings, 2020). In African continental series, available palaeoseasonality proxies (e.g., geochemistry of paleosol or mineralized tissues of fossil organisms, vegetal associations, dental wear) allow recording disparate information and are difficult to interpret because they depend on multiple factors (climate, ecology, physiology, geodynamics). New independent and complementary supports are needed to document the contrast between the dry and wet seasons in ancient times in Africa. Moreover, these new proxies must be abundant, and widely distributed in intertropical African outcrops dated from the Miocene. With this objective, we bet on using the fossil growth patterns of tropical freshwater ectotherm vertebrates, to track seasonal changes over their lifetimes.

Diet, temperature, and photoperiod (with the latest two often varying concomitantly) are usually mentioned to explain the seasonal fluctuation in ectothermic growth. They can induce a direct effect on the life of the animal, such as food abundance, or indirectly by indicating the upcoming future, and predictable conditions, such as temperature or the photoperiod (Gwinner, 1986).

The amount of food abundance in the environment is considered a key, direct, and limiting factor influencing circannual biological cycles, and in particular somatic growth, by positively controlling energy supply (Bayley, 1988; Henderson, 2005; Jones, 1986; Kerrigan, 1994; Mérona et al., 1988; Meunier et al., 1994; Rosenfeld et al., 2005; Welcomme, 1979; Xu et al., 2014).

In temperate regions, ambient temperature is an important factor in regulating ectothermic growth and might have a positive effect on metabolism (Castanet, 1978; Henderson, 2005). Some authors suggest the small seasonal variation in temperatures in the tropics would not explain growth rate variation (Bourlière, 1980; Mérona et al., 1988). But a shift in temperature as small as 2°C, a range observable in tropical regions between winter and summer, has been shown to produce a change in some fish's growth rate (Longhurst & Pauly, 1987; Pauly & Ingles, 1981). For instance, water temperature strongly regulates somatic growth: in *Oreochromis niloticus*, for which growth is almost twice greater at 28°C (weight gain of 0.02 g/day) than at 24°C (0.01 g/day) or 32°C (0.1 g/day) after 50 days (El‐Sayed, 2006) and in southeastern Asian fish (Islam et al., 2019; Lowe‐McConnell, 1987; Welcomme, 1979).

Finally, photoperiod (day length) is considered to be the most important environmental parameter, regulating many biological functions in animals (reproduction, development, food intake and efficiency, locomotor activity, food intake, etc.; Boeuf & Le Bail, 1999). It is also generally positively correlated with somatic growth in fish as demonstrated in some tropical fish species such as *Oreochromis niloticus*, for which feed efficiency is also increased under long day length (Biswas et al., 2004; Biswas & Takeuchi, 2003; El‐Sayed & Kawanna, 2007; Veras et al., 2013) or *Colossoma macropomum* (Mendonça et al., 2012). But, in some tropical fish species, the correlation has been reported to be negative, as in *Clarias gariepinus*, which shows increased growth in the complete night with short day length (Almazán‐Rueda et al., 2005; Mustapha et al., 2012), and in *Labeo rohita* where prolonged photoperiod negatively affected growth (Shahjahan et al., 2020). In some cases, no photoperiod effect has been identified, as in the catfish *Lophiosilurus alexandri* (Kitagawa et al., 2015) or the Chinese softshell turtle *Pelodiscus sinensis* (Li et al., 2020; Xianqing et al., 1999).

Other factors that may control the growth of ectotherms are multiple, and besides environmental factors, also depend on the ecology, development, and phylogeny (e.g., Britton et al., 2011; Britz & Pienaar, 1992; Castanet et al., 1992; Johnson & Dick, 2001; Limpus & Chaloupka, 1997; Martin‐Smith & Armstrong, 2002; Mérona et al., 1988; Rosenfeld et al., 2005; Toguyeni et al., 2002). In addition, fewer data are available in the literature on tropical species, and even more so if they are not exploited for trade.

Fossil remains of freshwater ectotherm vertebrates are found in abundance in the intertropical African continental series and have recorded the growth rate of these animals in ancient times. However, our ability to extrapolate seasonal variation using growth observations from fossils relies on our knowledge of its multifactorial control in extant relatives. This knowledge is very fragmentary and sometimes nonexistent for extant species closely related to the intertropical African fossil taxa.

This study presents the results of an experiment, aiming at evaluating the influence on somatic growth of the three mentioned environmental parameters on three species of freshwater ectotherms, two fishes the bichir *Polypterus senegalus* and the catfish *Auchenoglanis occidentalis*, and the turtle *Pelusios castaneus*.

Fossil remains of these vertebrates are abundant in intertropical African continental series since the Miocene, especially in well‐studied sites of Chad and Eastern Africa, in which seasonality remains to be investigated. This experiment will serve as a reference for the relationship between growth rate and seasonality to study the evolution of seasonality in ancient African contexts.

For 1 year, replicate batches were subjected to simulated seasonal variation in food abundance, water temperature, or photoperiod (only for the two species of fish), similar to what wild populations would be expected to experience in situ, while these parameters were kept constant in Control groups to answer the following questions:

1. Does the variation of the food abundance or water temperature, mimicking the alternation of fluctuating conditions during wet and dry seasons, or photoperiod induce change in growth rate in the three studied freshwater ectotherms, and to what extent?
2. Does the prolongation of stressful conditions over 3 months, mimicking the short or long dry season, induce a change in growth rate across time, such as a progressive stop in growth?

As we observed in this experiment other, uncontrolled, factors have an impact on growth, we include a discussion about the possible causes identified: the presence of compensatory growth, internal growth rhythm, and social status.

## 2 MATERIALS AND METHODS

### 2.1 Study species

Three tropical freshwater vertebrate species were chosen for their abundance in the African fossil record since the early Miocene: the fishes *Polypterus senegalus* (Gray bichir or Cuvier's bichir) and *Auchenoglanis occidentalis* (Bubu or Giraffe catfish), and the turtle *Pelusios castaneus* (Side‐neck turtle or West African mud turtle). Their fossil remains are abundant (notably scales of *Polypterus* sp., thorny rays of catfish fins, and carapace plates of *Pelusios* sp.) in sites of Central and Eastern Africa since the Miocene, and they can be attributed at least at the generic level, even when found isolated (e.g., Garcia, unpublished; Otero, unpublished).

*Polypterus senegalus* is found in lakes, river margins, swamps, freshwater lagoons, and flood plains of tropical West Africa, the Nile Basin, and lakes Turkana and Albert (Gosse, 1990). The habitat preference of the bichir is swampy or muddy waters (Froese & Paugy, 2019), and temperatures range from 25 to 28°C (Baensch & Riehl, 1985). This predator preys mainly on insects, fishes, crustaceans, mollusks, frogs, and worms, and also feeds on plants and seeds (Ayoade et al., 2018). Sexual maturity is reached within its first year of life, and the growth rate of females is higher than that of males (Daget et al., 1965).

*Auchenoglanis occidentalis* is found in the shallow waters with muddy bottoms of the Congo, Nile, Omo, and Jubba rivers, and in the Lakes Tchad, Turkana, Albert, and Malawi (Ikongbeh et al., 2014; Risch, 1990; Verbeke, 1959). This catfish eats mainly insects or insect larvae and algae. A seasonal rhythm in feeding has been noticed since stomachs are found empty more frequently during the dry season than during the wet season (Ikongbeh et al., 2014). Its habitat preference is turbid waters. It hides in shelters during the day and is more active at night (Froese & Paugy, 2019).

*Pelusios castaneus* is known in West Africa, from Senegal (suspected in Mauritania) to the Central African Republic, Angola, and in the Sudanese and Guinean savannas (Rhodin et al., 2017; Trape et al., 2012). It inhabits shallow permanent or semi‐permanent bodies of fresh‐to‐brackish water. This turtle is omnivorous, feeding on invertebrates, amphibians, carrion, and plants. It estivates during the dry season in the mud and vegetation (Trape et al., 2012).

### 2.2 Study design

#### 2.2.1 General design

The experiment was housed by the “Ecologie, Evolution, Symbiose” (EES) team from the UMR CNRS 7267—Ecologie et Biologie des Interactions (EBI). It was followed by the C2EA 45‐COMETHEA Poitou‐Charentes, approved by the French Ministry of Higher Education and Research under the ethical agreement number 86‐050. The experiment was monitored by a veterinary service to attest to the well‐being of the animals during the experiment. Specimens belonging to the same cohort were bought from the same animal farm: Ferme Tropicale, Paris for turtles, and Aquaterra‐Diffusion for fishes. The 20 specimens of turtles (Togo) and the 23 specimens of catfish (Cameroon) were known to have been captured in the wild, while the 25 specimens of bichir were reared on an Indonesian farm (Table 1).

**TABLE 1:** Summary of growth data for the individuals from each treatment group of the experiment.

Treatment groups	Number of individuals	Mean initial length (cm)	Mean final length (cm)	Mean initial weight (g)	Mean final weight (g)
*Polypterus senegalus*					
Control	9	7.38	11.04	2.76	11.97
Resource	5	7.41	9.48	2.88	7.08
Temperature	5	7.06	9.56	2.74	7.84
Photoperiod	6	7.70	10.93	3.017	10.76
*Auchenoglanis occidentalis*					
Control	9	10.65	18.65	19.64	121.13
Resource	5	10.52	18.12	18.08	105.16
Temperature	4	9.63	17.82	18.98	102.73
Photoperiod	5	10.34	17.49	16.74	85.87
*Pelusios castaneus*					
Control	6	10.18	11.84	179.73	298.32
Resource	6	9.79	11.50	168.80	274.52
Temperature	8	10.35	12.92	194.68	383.66

*Note*: A summary for each individual is available in the Dryad (https://doi.org/10.5061/dryad.9w0vt4bkw). For fishes, the length corresponds to the standard length and to the carapace length for the turtles. Final length and weight for the *A. occidentalis* are those of the seventh month, before the death event.

**FIGURE 1:**
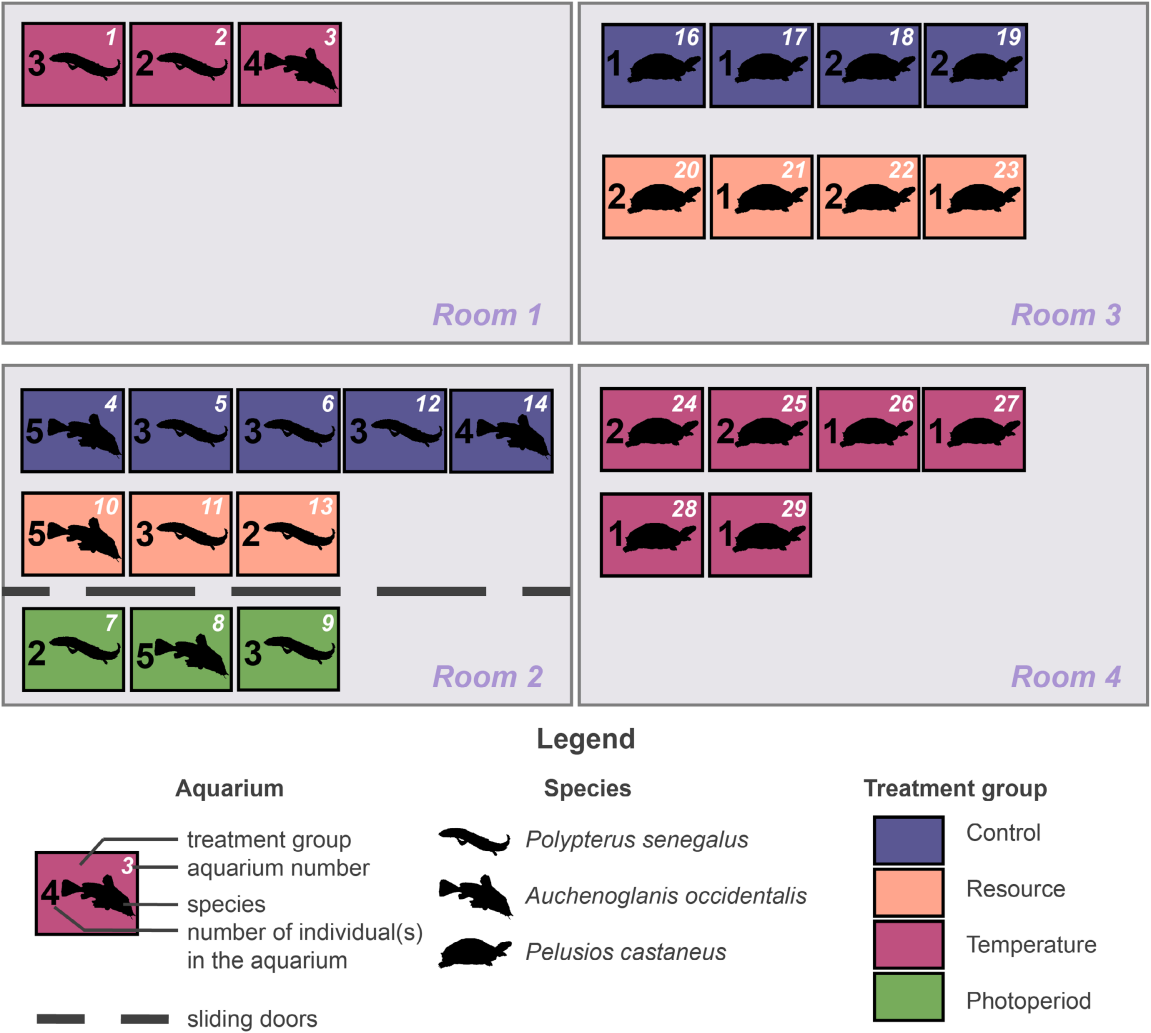
Distribution of specimens between aquariums and treatment groups, and aquariums in rooms with individual water circuits.

The experiment took place in four independent rooms (Figure 1). Each of the rooms was equipped with an independent water circuit with a tank. The water temperature in each tank was regulated by two 300 W immersion heaters, with an accuracy of 0.5°C. The tanks were equipped with filters composed of a layer of polyurethane foam at the bottom, just above the cooling circuit, surmounted by four bags of 10 to 15 kg of pozzolan, which was regularly cleaned when dirty. Each tank received a dose of 10 mL per 40 L of Aqua Quick Start (API®) water for the nitrogen cycle. The water was renewed regularly thanks to a pump with a capacity of 2400 L/h, allowing regular oxygenation of the water.

Each room was lit by three independent and programmed fluorescent growth light bars (OSRAM L 36W/77 Fluora), and the lighting provided for the turtles included 10% UVB, necessary for their development. The experiment was carried out in 28 aquariums of 40 L (L.55 × W.40 × H.20 cm), distributed in the four rooms (Figure 1). Two rooms were used for fish (1 and 2), and two more for turtles (3 and 4; Figure 1). Rooms 2 and 3 housed the Control, Resource, and Photoperiod (only for the two species of fishes) aquariums while Rooms 1 and 4 housed the Temperature tanks (see description of the groups at the end of the paragraph; Figure 1). Room 1 was equipped with upper and lower shelves. The fishes from the Control groups were distributed over the two shelves. The fishes from the Photoperiod groups were on the top shelf, just under the lights. This shelf is separated from the others thanks to sliding doors allowing light insulation, therefore allowing to apply its photoperiod. The fishes from the Resource group were placed on the lower shelf. In the other rooms, the aquariums are all on the top shelf near the lights. There were 2 to 3 bichirs per aquarium or 4 to 5 catfishes or 1 to 2 turtle(s) (Figure 1). At the end of the experiment, the density of individuals per aquarium did not show any statistically significant effect on the growth rate of animals (linear mixed‐effect models, *p* > .05; Figure 1). Moreover, animals distributed in groups have statistically similar length and weight at the beginning of the experiment, and at the end of the experiment (ANOVAs, *p* > .05; Table 1).

Three times a week, the aquariums were cleaned with an aquarium vacuum and a landing net. Also, the volume of water changed varied over time and from room to room. Before reinjection into the circuits, the water was stored in a heated tank, and received 10 mL per 40 L of water from the water conditioner. For fishes' water, a third of the changed water was replaced by demineralized water since they are sensitive to water hardness.

To cover the needs of each species, turtle aquariums were equipped with a ceramic heating lamp (connected to a thermostat) above a basking area, below which there is a submerged and shadowed shelter, as well as a semi‐emerged shelter (PVC pipe). The walls were opaque and surrounded by plastic grids to prevent falls. The fishes' tanks were equipped with an opacified cover, to reproduce the deeper and turbid waters of their natural environment, and with half‐PVC pipes serving as shelters. Over time, these hiding places were replaced with larger ones adapted to the growing size of catfishes. The feeding schedule and the ad libitum diet amount were established for each species during the acclimatization phases, then reassessed throughout the experiment based on food remaining 2 h or 1 day after meals for fish and turtles, respectively. To cover their needs, the animals received a varied diet spread over weekly cycles. The fish diet was composed of flakes (TetraMin® for tropical fish, and TetraMin Pro Crisps) and insect *Chironomus* sp. larvae, while the turtles were fed with Zoo Med® Aquatic Turtle Food (Growth formula), raw beef liver every 2 weeks, and a cuttlefish bone to provide calcium.

**FIGURE 2:**
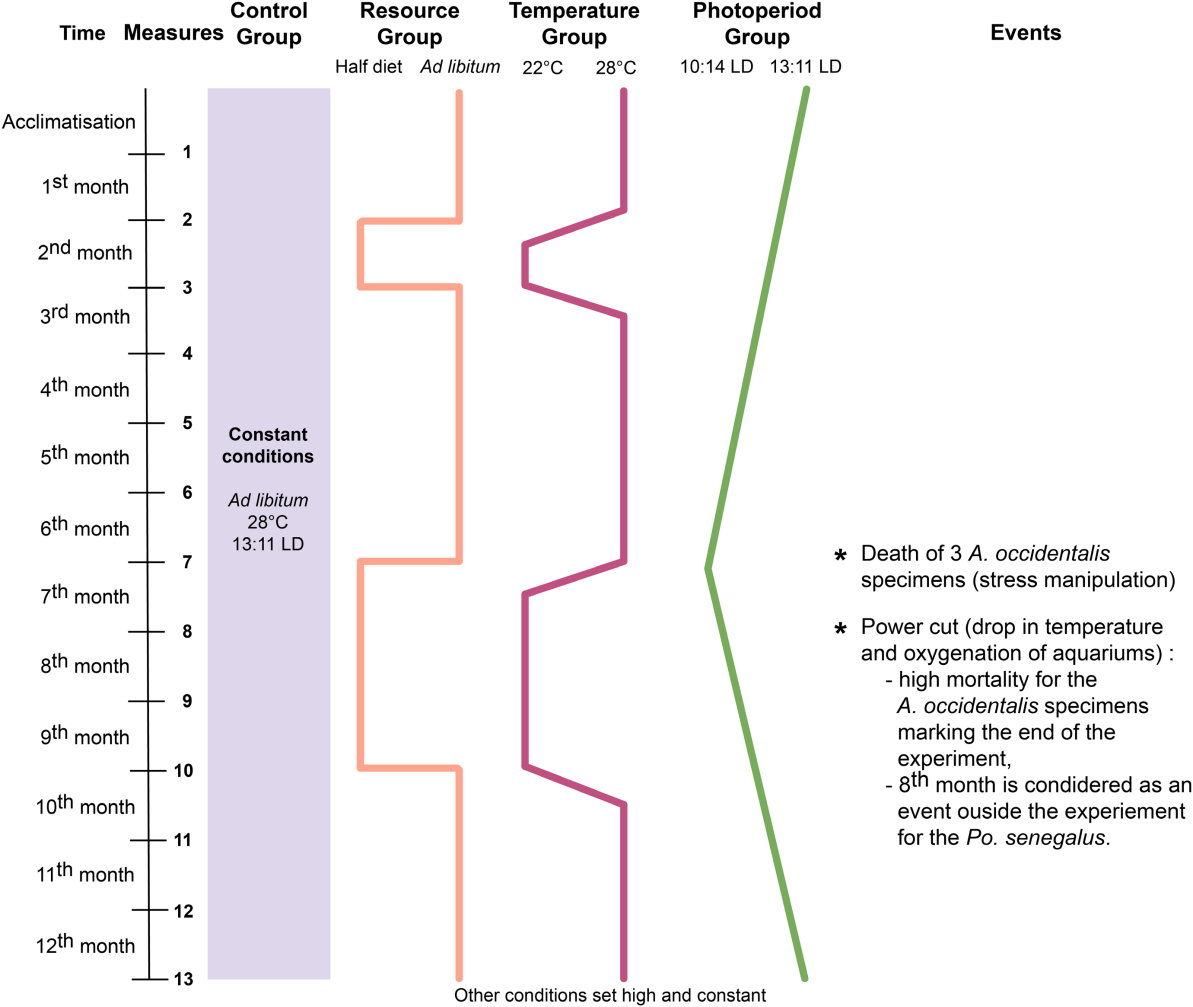
Experimental conditions applied to the different treatment groups and relevant events over time. Animals were fed to apparent satiation (ad libitum) or were fed with half of the ad libitum diet amount (half diet). The Photoperiod group concerns only fishes. 13:11 LD means that animals were reared under a regime of photoperiod of 13 h of light and 11 h of dark per day. *Events refer to experimental issues encountered.

The treatments imposed on the animals were divided into two modalities, referred to hereafter under the terms of “high conditions,” where the animals were fed ad libitum and/or the water in the aquarium was at 28°C and/or the length of the day was greater than 11 h, or “low conditions,” where the animals were starved and/or the water temperature was 22°C and/or the length of the day was less than 11 h (Figure 2). During the experiment, short and long phases of “high” or “low” conditions alternate, to identify the effect of the variation of these parameters on the growth rate of the animals (Figure 2). Feeding at apparent satiety or ad libitum made it possible to set a benchmark for a nonstressful condition for the animals in the context of the experiment, which can be easily reproduced. Furthermore, this is a condition that can be reached at certain times of the year in a natural environment, for example during the wet season (Gwinner, 1986; Lowe‐McConnell, 1987). To simulate stress, the quantity of food to satiety was, during periods of “low conditions,” halved, allowing not to completely starve the animals but having a range of food abundance large enough to hope to see effects of this decrease on growth. The variation in water temperature between 22 and 28°C corresponds to the range of average temperatures recorded in Chad during the year (Merkel, 2020; Roche, 1971). The chosen photoperiod range is from 13:11 light–dark (LD) to 10:14 LD. This photoperiod variation corresponds to that of Egypt, which is the northern limit of the current repartition of *Po. senegalus* and *A. occidentalis*. Again, expecting to observe the effects of the photoperiod on the growth rate, we have chosen a large range of photoperiod, in Egypt where the amplitude of photoperiod is greater than that in Chad (3.5 h instead of 1.5 h in Chad).

Individuals of each species were separated into 3 to 4 groups (Table 1), which received different treatments:

1. In the Control group, the animals were subjected to constant parameters during the whole of the experiment and to all the high conditions, expected to be experienced by animals in the wild during the summer or the wet season: an apparent satiety feeding or ad libitum, water at 28°C and a 13:11 LD photoperiod (Figure 2).
2. In the Resource group, the animals were subjected for 1 month, then after 4 months again for 3 months to low conditions, during which the amount of food provided was divided by two to simulate starvation (mimicking dry season; Figure 2). The rest of the time the animals were fed at satiation (high conditions, mimicking wet season; Figure 2). Other conditions were high and set constant (Figure 2).
3. The Temperature group was submitted to two low‐condition periods of water temperature decreasing to 22°C (for 1 month, and then, 4 months later, over three consecutive months; Figure 2). The temperature change was gradual and carried out over 2 weeks. The rest of the time the temperature was maintained at 28°C (Figure 2). Other conditions were high and set constant (Figure 2).
4. The Photoperiod group only concerns both species of fish (Figure 1). The effect of the photoperiod has not been tested on turtles for reasons of well‐being: the conditions were already particular and the environment had to be at least viable, providing the necessary lighting. At the beginning of the experiment, the photoperiod regimen for this group was 13:11 LD (Figure 2). The length of the day was dimmed by 7 min every week until reaching a photoperiod of 10:14 LD in the middle of the experiment (Figure 2). The length of the day was then further increased by 7 min each week, again reaching 13:11 LD at the end of the experiment (Figure 2). Other conditions were high and set constant (Figure 2).

Every change applied to environmental conditions was accompanied by an injection of a calcium‐binding fluorescent dye. Temperature and pH of the water were measured daily, the water quality parameters, were weekly checked, and finally, somatic growth (standard length of fishes and carapace length of turtles) and weight of each specimen were measured monthly. At the end of the experiment, the animals were euthanized by an overdose of anesthetic.

### 2.3 Course of the experiment

**FIGURE 3:**
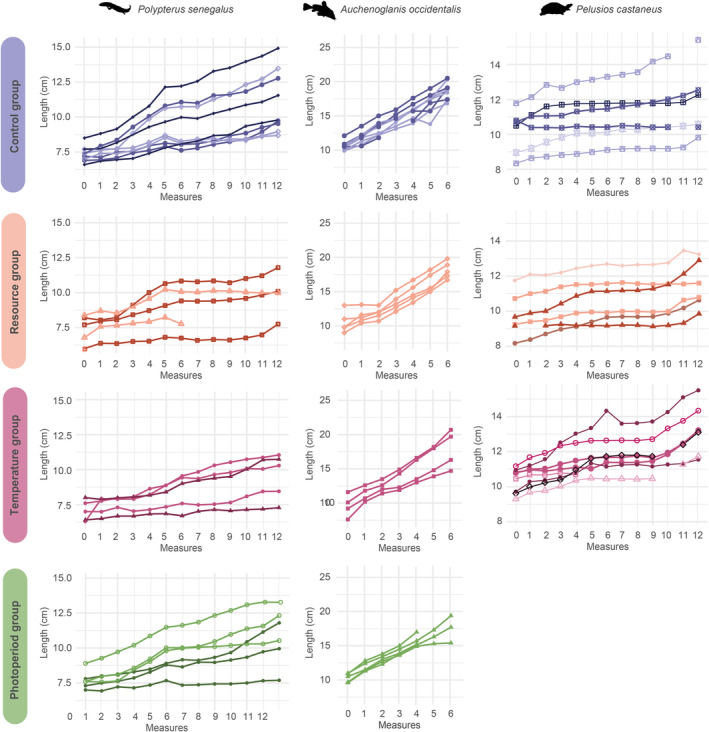
Growth in length of each specimen during the experiment. For each species, individuals bred in the same aquarium have the same color and the same symbol.

Fishes and turtles in the Control group do not show any inflection in growth (in the Control group): the growth is still linear (Figure 3). At the end of the experiment, they are probably at the very beginning of the allocation of energy resources to reproduction (and not to growth). Fishes are below a quarter of the maximum size of their respective species, for some the sexual differentiation was not visible (Froese & Paugy, 2019).

Social hierarchy marks were observed in the aquariums of *Po. senegalus* only, generally in favor of the larger specimens. This hierarchy resulted in mutilations inflicted on small specimens (damage on the caudal fin, punctured eye), which were absent in the largest specimens. Our observations did not allow us to identify the social status of each individual and to analyze this aspect in more detail.

Between the seventh and eighth months of the experiment, a power cut caused the pumps to stop, resulting in a rapid drop in water temperature (decrease of 4 to 10°C) and oxygenation for less than 24 h (Figure 2). This event had very visible repercussions; in particular, high mortality among *A. occidentalis*, and could also have involved stress in *Po. senegalus*, which was visible in the measurements of the following (eighth) month. The animals lost their appetite for a week before returning to normal. We slightly increased the amount of food distributed 1 week after the incident. This experiment has shown that *A. occidentalis* were very sensitive to rapid environmental variation, particularly those concerning water quality (pH, parasites, temperature, phosphate concentration). This event and periods of variation in water quality were considered to be the causes that led to the death of more than 70% of the *A. occidentalis* of the experiment by the seventh month.

The growth data of *A. occidentalis* are not presented in the article after the sixth month, as well as for the *Po. senegalus*' eighth month.

### 2.4 Measurements

Each month, the animals were not fed during the day before the measurements. As for the two species of fish, they were anesthetized in a bath of methanesulfonate (TM or MS‐222) diluted to 100–110 mg/L in water taken from their box. The water in the manipulation tank is continuously oxygenated, and the temperature is maintained using a 60 W immersion heater. Turtles were handled without anesthesia, which could have threatened their health. The measurements of the standard length for fishes and carapace length for turtles were carried out with an electronic caliper with an accuracy of one‐hundredth of a centimeter. The weighing with an electronic balance with an accuracy of the gram. One operator carried out most of the measurements, but he was temporarily replaced by one different operator a few times.

Negative animal length growth rate values have been calculated several times but are unrelated to operator changes. These negative values constitute 14.6% of the bichir measurements, 2.6% of the catfish measurements, and 16.2% of the turtle measurements. For some animals, growth was so slow that the size of the animals from month to month was very similar, if not the same. A priori, it is assumed that the constituents of the skeleton cannot be remobilized, and the animal's length cannot be reduced, unlike fat reserves or musculature (Broekhuizen et al., 1994). Therefore, negative growth rates could also be due to the low monthly growth of individuals, combined with the contraction or relaxation of animals' bodies, which could affect measurements. However, as it is not possible to know whether the first measurement was overvalued or the second one undervalued, we chose to keep all the values except those which do not relate to a biological phenomenon but an obvious measurement error.

### 2.5 Data analysis

The growth rates recorded for *Po. senegalus* in the eighth month and the growth rates of *A. occidentalis* after the sixth month were not considered for the reasons explained in Section 2.3.

To compare growth rates over short periods (~1 month), and since the animals of the same species were of similar age (similar size, so similar life stage), the *specific growth rate* (SGR) was the most suitable way to express growth in this experiment (Hopkins, 1992; Lugert et al., 2014). SGR is expressed as the percentage increase in weight (or length) per day (Hopkins, 1992; Lugert et al., 2014). We calculated the rate for both weight and size. For the weight, $\mathrm{SGR}=\frac{\ln\left(\mathrm{wt}\right)-\ln\left(\mathrm{wi}\right)}{t}\times 100$ where *wt* is the weight at the end of the month, *wi* is the weight at the beginning of the month, and *t* is the duration of the period in days. In the following, we used the abbreviations SGRW for the specific growth rate of weight and SGRL for the specific growth rate of length.

Mean growth rates were expressed as the sample mean ± standard error. The significance of statistical test results was evaluated at *α* = 0.05. All analyses were performed in R Version 4.2.2 (R Core Team, 2022), and figures were created in the ggplot2 package (Wickham, 2016).

The effect size is also provided and expressed as the Hedges' *g* and its 95% confidence interval to quantitatively compare the results. The difference between the two samples is large when *g* > 0.8, medium when 0.5 < *g* < 0.8, and small when 0.2 < *g* < 0.5.

#### 2.5.1 Effect of variation in controlled environmental parameters on growth rate

We fitted linear mixed‐effects models (*nlme* package; Pinheiro et al., 2023) to predict SGRL or SGRW with Resource, Temperature, or Photoperiod. The model included the specimens and tanks as random effects. When autocorrelation was detected in the model, we applied an AR1 correction. For each model, we considered data from the corresponding Treatment and Control groups. Establishing a model directly linked to the quantity of food (even as a ratio to the weight of the animal) does not give significant results, because the quantity of food to reach ad libitum evolves exponentially as a function of the mass of the animals and therefore over time. For this, we preferred to use a categorical variable (ad libitum feeding vs. starvation or half ad libitum) as an independent variable of our models linked to food abundance.

#### 2.5.2 Effect of the duration of low‐condition period on growth rate

We investigated the impact of the prolongation of low conditions over 1 to 3 months, mimicking the short or long dry season, on the growth rate. Here, we want to check (1) whether even short seasonal changes can affect the growth rate of these animals and (2) whether the prolongation of low conditions can slow or even stop growth over the course of months.

We used linear mixed‐effects models (*lme4* and *lmerTest* packages; Bates et al., 2015; Kuznetsova et al., 2017). The models include the specimens and tanks as random effects. Significant differences in growth rate between months were identified by post hoc Tukey's test with a Bonferroni correction (*multcomp* package; Hothorn et al., 2016). For the reasons given earlier, this part only concerns bichirs (excluding the eighth month) and turtles but not catfish because they only have reliable data for 1 month of low conditions. Thus, we aimed to identify growth rate differences between the second and seventh (1‐month duration), eighth (2‐month duration), and ninth (3‐month duration) months of the experiment for the Resource and Temperature groups. For bichirs from the Photoperiod group, we examined the fifth (1‐month), the sixth (2‐month), and seventh (3‐month) months of the experiment.

#### 2.5.3 Compensatory growth phases

Compensatory growth is a period of increased growth rate following a period of low conditions, after the re‐establishment of high conditions (Ali et al., 2003; Jobling, 2010; Oksbjerg & Therkildsen, 2017). This can exceed the usual growth rate during favorable living conditions and could result in the restoration of the normal growth trajectory (Ali et al., 2003; Broekhuizen et al., 1994; Jobling, 2010; Oksbjerg & Therkildsen, 2017). We aim to highlight the compensatory growth phase: (1) by comparing the growth rate of the Control group with that of a treatment group 2 months after a low‐condition period (third, fourth, eleventh, and twelfth months for the Resource and Temperature groups, and months 9 and 10 for the Photoperiod group), and (2) by comparing the growth rate within a treatment group between a low‐condition period (mean growth rate by an individual if several months) and the two following months. For (1), The growth rate between the Control group and Temperature or Resource groups was compared using linear mixed‐effects models (*lme4* and *lmerTest* packages; Bates et al., 2015; Kuznetsova et al., 2017) and pairwise post hoc multiple comparisons (*afex* and *emmeans* packages; Lenth & Lenth, 2017; Singmann et al., 2015). The model included the specimens and tanks as random effects. For (2), significant differences in growth rate between months were identified using linear mixed‐effects models (*lme4* and *lmerTest* packages; Bates et al., 2015; Kuznetsova et al., 2017) and post hoc Tukey's test with a Bonferroni correction (*multcomp* package; Hothorn et al., 2016). The model included the specimens and tanks as random effects.

#### 2.5.4 Other observations on the factors controlling the growth

Because this experiment should allow studying fossil data, it was essential to accurately describe the impact of controlled factors, and also that of the potential other, uncontrolled, factors to explain the observed signal. In this section, we qualitatively described and compared the mean growth rate of the different treatment groups, month by month, to identify general trends or events that were not highlighted by the previous statistical analyses.

## 3 RESULTS

There is no evidence that day length affected the growth rates of bichirs and catfish from the Photoperiod group during the experiment. Moreover, the observed growth rate fluctuations are very similar to those observed in the Control group. Results corresponding to the Photoperiod group are presented in Dryad (https://doi.org/10.5061/dryad.9w0vt4bkw).

### 3.1 Effect of variation in controlled environmental parameters on growth rate

#### 3.1.1 Po. senegalus

Growth rates of bichirs are positively affected by food abundance and temperature (Table 2). Reducing the amount of food by half during low‐condition periods induces a drop of 0.08%/day (0.03–0.14) of the SGRL and 0.59%/day (0.44–0.74) of the SGRW. A 1°C reduction water temperature induces a decrease of 0.01%/day in SGRL (0.00–0.02) and of 0.04%/day (0.01–0.08) of the SGRW (Table 2).

**TABLE 2:** Parameter estimates with 95% confidence interval in brackets and standard error linear mixed‐effects models for *Polypterus senegalus* specimens.

*Polypterus senegalus*	Specific growth rate of the length (SGRL)			Specific growth rate of the weight (SGRW)		
Predictors	Coefficient estimate	Standard error	*p*	Coefficient estimate	Standard error	*p*
Food abundance (1)	**0.08 (0.03–0.14)**	**0.03**	**.00**	**0.59 (0.44–0.74)**	**0.08**	**.00**
Water temperature (2)	**0.01 (0.00–0.02)**	**0.01**	**.04**	**0.04 (0.01–0.08)**	**0.02**	**.01**

*Note*: Significant effect is reported in bold. Models include specimens and tanks as random effects. (1) Modeling growth rate as the function of conditions in food abundance (starvation vs. ad libitum) of the Resource and Control groups' specimens; (2) modeling growth rate as the function of water temperature of the Temperature and Control groups' specimens.

Abbreviation: *p*, *p*‐value.

#### 3.1.2 A. occidentalis

Growth rates of catfishes are positively affected by food abundance (Table 3). Reducing the amount of food by half during low‐condition periods induces a drop of 0.14%/day (0.00–0.27) of the SGRL and 0.58%/day (0.25–0.92) of the SGRW. However, water temperature fluctuation does not significantly affect growth rates (Table 3).

**TABLE 3:** Parameter estimates with 95% confidence interval in brackets and standard error linear mixed‐effects models for *Auchenoglanis occidentalis* specimens.

*Auchenoglanis occidentalis*	Specific growth rate of the length (SGRL)			Specific growth rate of the weight (SGRW)		
Predictors	Coefficient estimate	Standard error	*p*	Coefficient estimate	Standard error	*p*
Food abundance (1)	**0.14 (0.00–0.27)**	**0.07**	**.05**	**0.58 (0.25–0.92)**	**0.17**	**.00**
Water temperature (2)	0.01 (−0.03–0.06)	0.02	.50	0.07 (−0.02–0.17)	0.05	.12

*Note*: Significant effect is reported in bold. Models include specimens and tanks as random effects. (1) Modeling growth rate as the function of conditions in food abundance (starvation vs. ad libitum) of the Resource and Control groups' specimens; (2) modeling growth rate as the function of water temperature of the Temperature and Control groups' specimens.

Abbreviation: *p*—*p*‐value.

#### 3.1.3 Pe. castaneus

The growth rates of turtles are positively affected by food abundance and temperature (Table 4). Reducing the amount of food by half during low‐condition periods induces a drop of 0.04%/day (0.02–0.04) of the SGRL and 0.11%/day (0.04–0.18) of the SGRW, as well as a 1°C reduction water temperature, induces a decrease of 0.02%/day in SGRL (0.01–0.02) and of 0.02%/day (0.01–0.03) of the SGRW (Table 4).

**TABLE 4:** Parameter estimates with 95% confidence interval in brackets and standard error linear mixed‐effects models for *Pelusios castaneus* specimens.

*Pelusios castaneus*	Specific growth rate of the length (SGRL)			Specific growth rate of the weight (SGRW)		
Predictors	Coefficient estimate	Standard error	*p*	Coefficient estimate	Standard error	*p*
Food abundance (1)	**0.04 (0.02–0.04)**	**0.01**	**.00**	**0.11 (0.04–0.18)**	**0.03**	**.00**
Water temperature (2)	**0.02 (0.01–0.02)**	**0.00**	**0**	**0.02 (0.01–0.03)**	**0.01**	**.00**

*Note*: Significant effect is reported in bold. Models include specimens and tanks as random effects. (1) Modeling growth rate as the function of conditions in food abundance (starvation vs. ad libitum) of the Resource and Control groups' specimens; (2) modeling growth rate as the function of water temperature of the Temperature and Control groups' specimens.

Abbreviation: *p*, *p*‐value.

### 3.2 Effect of the duration of low‐condition period on growth rate

#### 3.2.1 Po. senegalus

The only significant difference identified for the bichirs during the low‐condition period concerned the Temperature group, with a greater SGRW after 1 month than after 3 months in water at 22°C (Δ = 0.28 ± 0.06%/day, *z* = −4.41, *p*‐value = 1.04e−05, *g* = 0.60 [−0.26, 1.54]; Table 5).

**TABLE 5:** Mean difference in growth rate ± standard error, during the consecutive months (duration) of low conditions (starvation or cool water) for *Polypterus senegalus* and *Pelusios castaneus*.

Species	*Polypterus senegalus*			*Pelusios castaneus*		
Group	Control	Resource	Temperature	Control	Resource	Temperature
	*n* = 9	*n* = 5	*n* = 5	*n* = 6	*n* = 5	*n* = 7
Δ Mean difference in specific growth rate of the length (SGRL) ± SE (%/day)						
1 versus 2 month(s) duration				0.04 ± 0.02	0.02 ± 0.01	0.01 ± 0.02
1 versus 3 month(s) duration	0.02 ± 0.03	0.00 ± 0.03	0.04 ± 0.03	0.02 ± 0.03	0.03 ± 0.01	0.01 ± 0.02
2 versus 3 months duration				−0.02 ± 0.02	0.00 ± 0.01	0.01 ± 0.01
Δ Mean difference in specific growth rate of the weight (SGRW) ± SE (%/day)						
1‐ versus 2‐month(s) duration				0.14 ± 0.06	0.15 ± 0.07	0.09 ± 0.04
1‐ versus 3‐month(s) duration	0.27 ± 0.12	0.06 ± 0.10	**0.18 ± 0.11**	0.12 ± 0.08	0.10 ± 0.08	**0.13 ± 0.06**
2‐ versus 3‐month duration				−0.01 ± 0.05	−0.05 ± 0.03	0.01 ± 0.04

*Note*: We aimed to identify growth rate differences between the second and seventh (1‐month duration), eighth (2‐month duration), and ninth (3‐month duration) months of the experiment for the Resource and Temperature groups. The growth rate of the 8th month is excluded from the analysis for *Polypterus senegalus* (see Section 2.3), therefore, we only compare the growth of the second + seventh and ninth months for this species. Values in bold: significant difference—*p*‐value ≤.05—between the mean growth rates recorded during the 2 months (linear mixed‐effects models and post hoc Tukey's tests); *n*—number of specimens per sample.

#### 3.2.2 Pe. castaneus

The only significant difference identified for the turtles during the low‐condition period concerned the Temperature group, with an SGRW greater after 1 month than after 3 months in water at 22°C (Δ = 0.13 ± 0.05%/day, *z* = −2.604, *p*‐value = .0247, *g* = 1.43, 95% IC [0.42, 2.62]; Table 5).

### 3.3 Compensatory growth phases

#### 3.3.1 Po. senegalus

In bichirs from the Resource group, the growth rate is greater during the 2 months following the return of ad libitum feeding (Table 6). However, during these months, the growth rate of the Resource group is not significantly greater than that of the Control group, thus rejecting the hypothesis of compensatory growth (Table 7).

**TABLE 6:** Mean difference in growth rate ± SE, between the low‐condition periods (starvation or cool water, during the second month for the first period, and seventh to ninth months for the second period) and the two following months after the return of high conditions (ad libitum feeding or warm water during the third and fourth months for the first period, and tenth and eleventh months for the second period).

Species	*Polypterus senegalus*			*Auchenoglanis occidentalis*			*Pelusios castaneus*		
Group	Control	Resource	Temperature	Control	Resource	Temperature	Control	Resource	Temperature
	*n* = 9	*n* = 4	*n* = 5	*n* = 9	*n* = 5	*n* = 5	*n* = 6	*n* = 5	*n* = 7
Δ Mean difference in specific growth rate of the length (SGRL) ± SE (%/day)									
2nd versus 3rd	−0.04 ± 0.06	**−0.12 ± 0.06**	0.09 ± 0.04	0.28 ± 0.07	**−0.19 ± 0.08**	0.07 ± 0.09	0.06 ± 0.04	−0.04 ± 0.03	**−0.05 ± 0.03**
2nd versus 4th	−0.06 ± 0.04	**−0.11 ± 0.06**	−0.03 ± 0.04	0.25 ± 0.06	−0.142 ± 0.068	−0.01 ± 0.05	0.03 ± 0.03	−0.03 ± 0.03	−0.03 ± 0.03
7‐9th versus 10th	−0.04 ± 0.03	**−0.06 ± 0.03**	**−0.10 ± 0.04**				0.00 ± 0.01	−0.03 ± 0.01	**−0.10 ± 0.02**
7‐9th versus 11th	**−0.06 ± 0.02**	**−0.07 ± 0.03**	−0.04 ± 0.04				−0.01 ± 0.01	**−0.12 ± 0.04**	**−0.12 ± 0.03**
Δ Mean difference in specific growth rate of the weight (SGRW) ± SE (%/day)									
2nd versus 3rd	0.07 ± 0.22	**−0.64 ± 0.17**	−0.09 ± 0.20	0.37 ± 0.12	**−0.93 ± 0.26**	0.18 ± 0.17	0.30 ± 0.10	0.37 ± 0.18	0.11 ± 0.07
2nd versus 4th	−0.08 ± 0.19	**−0.57 ± 0.20**	−0.25 ± 0.22	0.03 ± 0.13	**−0.79 ± 0.26**	−0.31 ± 0.19	0.17 ± 0.10	0.07 ± 0.12	−0.07 ± 0.08
7‐9th versus 10th	−0.12 ± 0.07	**−0.09 ± 0.10**	−0.25 ± 0.18				0.04 ± 0.05	**−0.10 ± 0.04**	**−0.14 ± 0.04**
7‐9th versus 11th	**−0.170 ± 0.06**	**−0.63 ± 0.21**	−0.03 ± 0.09				0.05 ± 0.03	**−0.10 ± 0.04**	**−0.20 ± 0.05**

*Note*: A higher growth rate after the return of high conditions (negative values in the table) may indicate a compensatory growth phenomenon. Values in bold: significant negative difference—*p*‐value ≤.05—between the mean growth rates recorded during the low‐condition periods and the two following months (linear mixed‐effects models and post hoc Tukey's tests); *n*—number of specimens per sample.

**TABLE 7:** Mean difference in growth rate ± SE, during the 2 months following the return of high conditions (ad libitum feeding or warm water) after low‐condition periods (starvation or cool water, after the second month and the ninth month) between treatment and control groups.

Species	*Polypterus senegalus*		*Auchenoglanis occidentalis*		*Pelusios castaneus*	
Groups	Resource versus control	Temperature versus control	Resource versus control	Temperature versus control	Resource versus control	Temperature versus control
Δ Mean difference in specific growth rate of the length (SGRL) ± SE (%/day)						
Δ 3rd month	0.00 ± 0.07	**−0.18 ± 0.06**	0.13 ± 0.06	−0.00 ± 0.09	**0.06 ± 0.02**	**0.07 ± 0.03**
Δ 4th month	−0.04 ± 0.07	−0.09 ± 0.04	0.05 ± 0.03	0.05 ± 0.04	0.02 ± 0.02	0.02 ± 0.02
Δ 10th month	−0.05 ± 0.03	0.05 ± 0.04			0.01 ± 0.02	**0.08 ± 0.02**
Δ 11th month	−0.05 ± 0.03	−0.03 ± 0.04			**0.10 ± 0.04**	**0.09 ± 0.03**
Δ Mean difference in specific growth rate of the weight (SGRW) ± SE (%/day)						
Δ 3rd month	−0.02 ± 0.23	**−0.33 ± 0.15**	0.30 ± 0.10	−0.24 ± 0.13	−0.06 ± 0.15	0.09 ± 0.06
Δ 4th month	−0.08 ± 0.24	−0.31 ± 0.14	0.15 ± 0.09	0.03 ± 0.15	0.01 ± 0.06	**0.14 ± 0.08**
Δ 10th month	0.35 ± 0.11	0.16 ± 0.18			0.07 ± 0.06	0.12 ± 0.06
Δ 11th month	0.04 ± 0.21	−0.10 ± 0.09			0.08 ± 0.04	**0.19 ± 0.06**

*Note*: A higher growth rate in the Treatment group and the Control group after the return of high conditions may indicate a compensatory growth phenomenon. Values in bold: significant difference—*p*‐value ≤.05—between the mean growth rates recorded during the low‐condition periods and the 2 following months (linear mixed‐effects models and pairwise post hoc multiple comparisons); *n*—number of specimens per sample.

#### 3.3.2 A. occidentalis

In catfish from the Resource group, the growth rate is greater in the month(s) following the return of ad libitum feeding (Table 6). Again, the growth rate of the Resource group is not significantly greater than that of the Control group, precluding to identify compensatory growth (Table 7).

#### 3.3.3 Pe. castaneus

On the contrary, in turtles, we can clearly distinguish the phenomenon of compensatory growth. In the Resource group, during the 11th month, the SGRL is greater than during the previous starvation period (7th to 9th months; Δ = 0.11 ± 0.02%/day, *z* = −6.684, *p*‐value <1e−04, *g* = 1.24, 95% IC [−0.23, −2.43], Table 6) and greater than in the Control group (Δ = 0.08 ± 0.03%/day, *df* = 23.4, *t*‐ratio = −2.973, *p*‐value = .0067, *g* = −0.63, 95% IC [−1.28, −0.03], Table 7).

Also, the growth rate of turtles from the Temperature group is greater during the 3rd, 10th, and 11th months in the Control group, and during the low‐condition periods indicating a compensatory growth following the period of cooler water temperature (Tables 6 and 7). For the 3rd month, the SGRL is greater than in the Control group (Δ = 0.07 ± 0.03%/day, *df* = 20.6, *t*‐ratio = −2.367, *p*‐value = .0278, *g* = −1.23, 95% IC [−1.94, −0.50]), and during the previous low‐condition period (Δ = 0.05 ± 0.02%/day, *z* = −2.430, *p*‐value = .0401, *g* = −0.86, 95% IC [0.09, 1.71]; Tables 6 and 7). For the 10th month, the SGRL is greater than in the Control group (Δ = 0.08 ± 0.03%/day, *df* = 21.4, *t*‐ratio = −2.357, *p*‐value = .0280, *g* = −1.00, 95% IC [−1.67, −0.32]), and during the previous low‐condition period (Δ = 0.104 ± 0.03%/day, *z* = −4.037, *p*‐value = .000205, *g* = −1.55, 95% IC [−3.10, −0.31]; Tables 6 and 7). Then, during the 11th month, the SGRL is greater than during the low‐condition periods (Δ = 0.12 ± 0.03%/day, *z* = −4.818, *p*‐value <1e−04; *g* = −1.27, 95% IC [−2.61, −0.16]) and in the Control group (Δ = 0.09 ± 0.03%/day, *df* = 27.7, *t*‐ratio = −2.560, *p*‐value = .0162, *g* = −1.05, 95% IC [−1.71, −0.39]). The SGRW is also greater than in the Control group (Δ = 0.18 ± 0.07%/day, *df* = 28.0, *t*‐ratio = −2.507, *p*‐value = .0183, *g* = −0.83, 95% IC [−1.45, −0.19]), and the low‐condition period (Δ = 0.20 ± 0.04%/day, *z* = 5.419, *p* < 1e−04, *g* = −1.29, 95% IC [−2.41, −0.33]; Tables 6 and 7).

Also, an ANOVA revealed no significant difference in length or weight at the end of the experiment depending on treatment groups, suggesting that even if the unfavorable conditions caused occasional declines in the growth rate, this delay was compensated by a relatively higher growth rate than in the control group during high‐condition periods in the three studied species.

### 3.4 Other observations on the factors controlling the growth

#### 3.4.1 Po. senegalus

**FIGURE 4:**
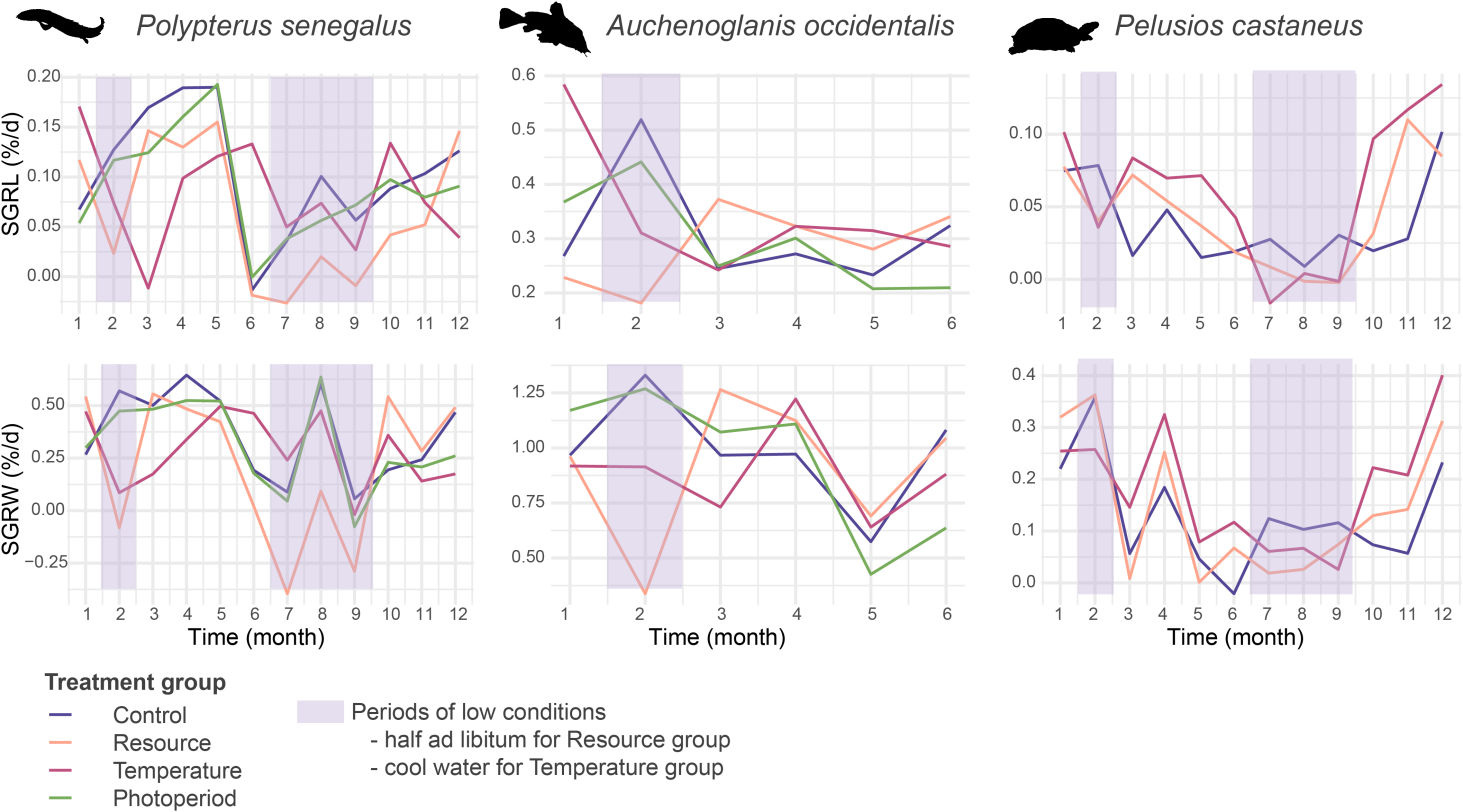
Average growth rates (SGRL and SGRW) in the different treatment groups of the three studied species during the experiment.

During the whole experiment, the Control group experienced constant conditions. However, the growth rate of *Po. senegalus* specimens in the Control group was far from constant (Figure 4). This implies a significant effect of a factor not controlled in the experiment playing an important role in the growth rate. The growth rates of *Po. senegalus* in the Control group and the Photoperiod group were very similar in both high and low conditions (Figure 4): the photoperiod regime did not affect the growth rate; however, the effect of the uncontrolled factor(s) mentioned above was again noticeable. The growth rate was high during the second to fifth months of the experiment, decreasing from the sixth to the ninth month, before increasing again (Figure 4). This trend was also observable to a lesser extent in the Resource group, for which the growth rate decreased during the sixth month, for reasons unrelated to the controlled factors (Figure 4). An abrupt increase in the growth rate for every group was recorded during the eighth month (Figure 4), which could be related to the brief stress induced by the stopping of the pumps during the preceding month, and leading to faster growth afterwards.

In general, one to three specimens per aquarium stood out from the others by their slightly larger size at the start of the experiment, and generally faster growth during the phases of high conditions, ranging from the third to the seventh months of the experiment. We notice that during the long low‐condition phases, specimens that we were able to identify as dominant showed a higher growth rate than the subordinates in the Temperature group, but the growth rate did not differ in the Resource group.

#### 3.4.2 A. occidentalis

The Control group showed a variable growth rate, suggesting the effect of uncontrolled parameters also in the case of the catfishes (Figure 4). There was a strong decrease in growth rate during the fifth month, which was also noticeable in other groups (Figure 4).

#### 3.4.3 Pe. castaneus

The growth rate of the Control group was variable while no low conditions were applied (Figure 4). Thus, other uncontrolled factors certainly affected the growth rate variation. The SGRW of *Pe. castaneus* in the Resource group was null during the third and fifth months (Figure 4). There was a brief increase in SGRW during the fourth month, which could be linked to the 9‐h stoppage of pumps in the aquariums.

## 4 DISCUSSION

### 4.1 Effect of food abundance, temperature, and photoperiod on growth rate of the three ectotherm species

#### 4.1.1 Food abundance

The reduction in food quantity resulted in a significant reduction in the growth rate of fishes and turtles. Thus, the available amount of food appeared to be the most important tested environmental parameter controlling growth in these tropical ectotherms.

#### 4.1.2 Temperature

The decrease from 28 to 22°C in water temperature, representing the temperature range that can be observed across the year in the African tropics, induced a significant reduction in the growth rate of *Po. senegalus* and *Pe. castaneus* but did not seem to affect *A. occidentalis*.

#### 4.1.3 Photoperiod

Only tested on fish, the effect of photoperiod variation on growth rate was not significant for the amplitude tested (range of 3 h over 1 year). Indeed, the growth rate evolution of the Photoperiod groups was very similar to that of the Control groups for both *Po. senegalus* and *A. occidentalis*, thus implying that the variation in the day length alone did not influence the growth rate of these tropical fishes.

Finally, two of the three tested parameters modified growth when varying within their natural range: (1) food abundance and (2) water temperature. Growth control by temperature underlines in *Po. senegalus* and *Pe. castaneus* that these animals have a narrow optimal temperature. Rainfall fluctuations and the resulting flooding and drying are the main elements of seasonality in tropical regions, playing a role in water temperature, oxygenation, turbidity, velocity, expansion or drying of freshwater areas, population density, and food abundance (Bayley, 1988; Lowe‐McConnell, 1987; Welcomme, 1979). Just after rainfall, it is mainly during the highwater period that the ectotherms breed, feed, and show very rapid growth (Gwinner, 1986; Lowe‐McConnell, 1987; Welcomme, 1979). The fat reserves made during this period allow survival during the dry season, when food is less abundant (Gwinner, 1986; Lowe‐McConnell, 1987).

### 4.2 Duration of low‐condition periods

According to our results for *Po. senegalus* and *Pe. castaneus*, the duration of the low‐condition periods did not influence the growth rate, which was lower than during the periods of high conditions but generally did not decrease further over time. These results show that a short period of 1 month of starvation or cooler water, mimicking a short season or periodic stress, is enough to produce a change in growth rate.

### 4.3 Compensatory growth

Following a period of low conditions (starvation, low temperature), the growth rate of turtles increased when conditions again become favorable, due to the compensatory growth phenomenon. This phenomenon is especially marked for the turtles of the Temperature group: they increased their growth rate after thermic stress compared with the Control group.

The high growth rate was recorded during the eighth month in all *Po. senegalus* (not considered in the analyses) may correspond also to a compensatory growth period in response to the low conditions (low temperature and deoxygenation) induced by the 24‐h power cut (Figures 2 and 3). Fish lost their appetite for several days but then showed a greater one so we had to sensibly increase the food quantities distributed to the bichirs to maintain ad libitum feeding. Then, the increase in the growth rate of the *Po. senegalus* specimens observed during the eighth month may correspond to a compensatory growth event in response to a short but intense period of stress.

### 4.4 Remarks on the possible uncontrolled factors that may explain growth rate during the experiment

#### 4.4.1 Social status

A difference in growth related to social status has already been shown in juveniles of *Salmo gairdneri* (Abbott & Dill, 1989), and juveniles of *Oncorhynchus mykiss* (Gilmour et al., 2005). The dominants grew faster than the subordinates with equal feeding (Abbott & Dill, 1989) or by the monopolization of food resources by the dominants (Gilmour et al., 2005), and mutilations in the fins were also observed in the subordinates. This difference in size has been attributed to the behavioral, metabolic, and physiological cost of stress, to a high cortisol level reducing appetite, mobilizing energy reserves, and to the cost of healing the injuries received, which thus contribute to a reduced growth rate among subordinates. At least for *Po. senegalus*, the social rank and maybe the density of population (to a greater extent than that in this experiment) could be an important factor controlling growth rate. Our observations did not allow us to identify the social status of each individual and to analyze this aspect in more detail.

#### 4.4.2 Internal rhythms

**FIGURE 5:**
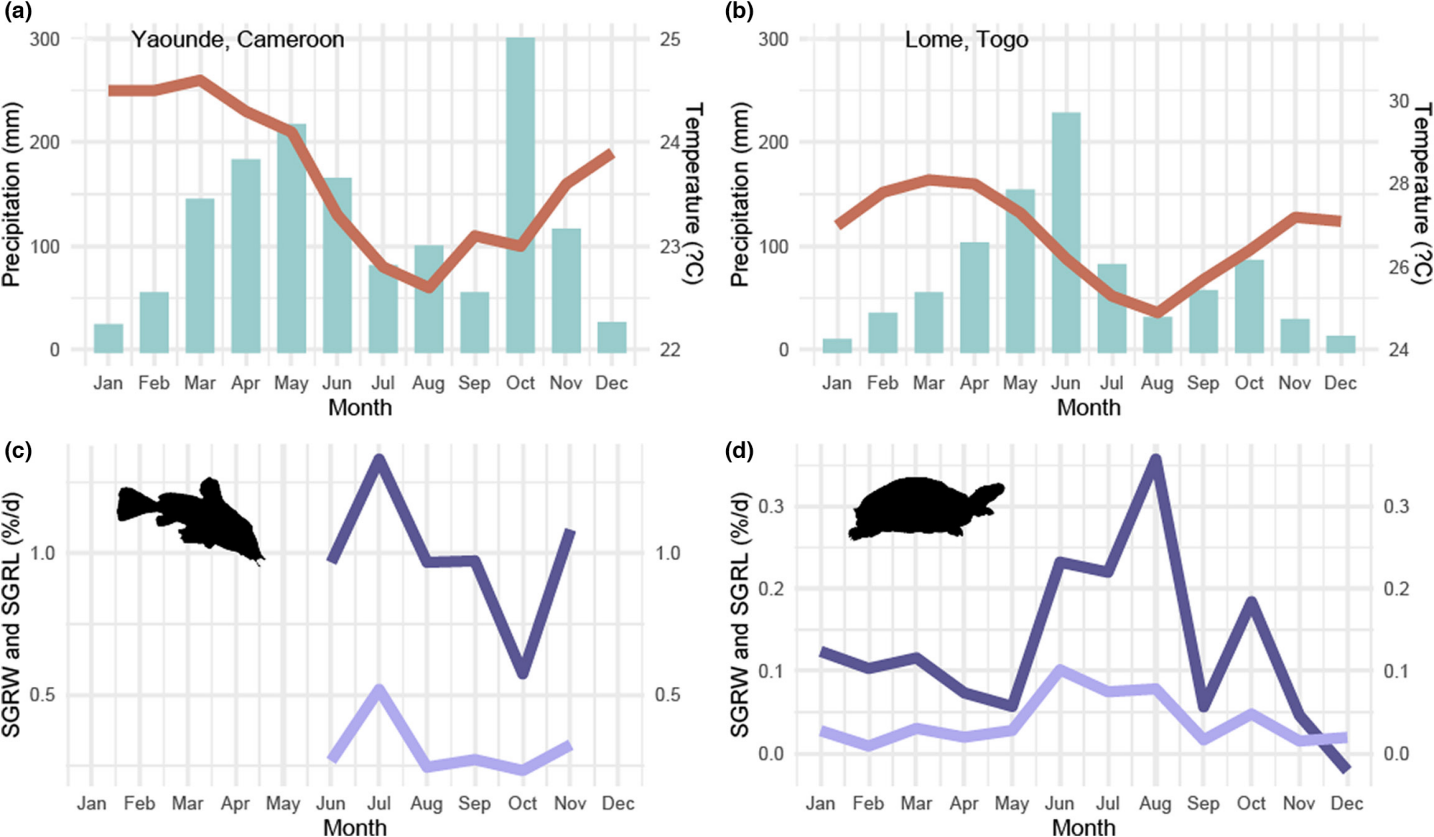
Cross‐referencing of growth data for *A. occidentalis* and *Pe. castaneus* reared under controlled conditions and climate data for three cities: (a, b) climate data (Merkel, 2020); (a) Yaounde in Cameroon (origin of *A. occidentalis* specimens); (b) Lomé in Togo (origin of *Pe. castaneus* specimens); (c, d) growth data in controlled conditions over the experiment with the light line representing mean monthly SGRW and the dark line representing mean monthly SGRL of the Control group; C: *A. occidentalis*; D: *Pe. castaneus*.

In the Control groups, the environmental conditions were kept constant for a year (with the only exception of the one stressful day of the power cut). However, the growth rate varied and followed a sinusoidal pattern. Indeed, fluctuations in growth rate seem to follow the seasons of the region of origin of the animals, suggesting that an internal rhythm might have controlled their somatic growth rate (Figure 5). The presence of an internal rhythm controlling biological factors has been demonstrated in both plants and animals living in temperate or tropical areas (Gwinner, 1986; Prokkola & Nikinmaa, 2018). This circannual rhythm allows organisms to anticipate, and adapt to specific seasonal environmental conditions in response to environmental stimuli such as the ambient temperature or the photoperiod (Gwinner, 1986; Prokkola & Nikinmaa, 2018).

## 5 CONCLUSION

Finally, this experiment highlights the close link between the growth rates of three tropical freshwater ectotherms and the seasonal variation in environmental factors. These species belong to genera abundant in the African fossil record since the early Miocene. Therefore, improved knowledge of their growth rate and ecology would allow the extrapolation of seasonal environmental variation in ancient times through the skeletal growth recorded in their fossil remains.

This experiment provides the first clues for interpreting the growth rate of fossil ectotherms closely related to the three studied species. Fluctuations in the growth rate of fossil taxa closely related to *Polypterus senegalus* and *Pelusios castaneus* could be interpreted in terms of the seasonality of water temperature and rainfall and flooding (that provide access to food resources). For fossil taxa close to *Auchenoglanis occidentalis*, variations in growth rate seem to be linked to seasonal variations in rainfall and flooding. Likewise, these fossil taxa would probably not be sensitive to variations in photoperiod during the year. Finally, the duration of application of low conditions did not modify the growth rate, so one would expect that short and long seasons would be recorded in the same way in the skeleton of these fossil ectotherms.

## AUTHOR CONTRIBUTIONS

**Axelle Gardin:** Conceptualization (equal); data curation (equal); formal analysis (lead); investigation (equal); methodology (equal); software (lead); validation (equal); visualization (lead); writing – original draft (lead); writing – review and editing (lead). **Olga Otero:** Conceptualization (lead); data curation (equal); investigation (equal); methodology (equal); project administration (equal); supervision (lead); validation (equal); writing – review and editing (equal). **Elodie Réveillac:** Validation (equal); writing – review and editing (equal). **Alexandra Lafitte:** Data curation (equal); investigation (equal); methodology (equal); project administration (equal); resources (lead); validation (equal); writing – review and editing (equal). **Xavier Valentin:** Investigation (equal); methodology (equal); writing – review and editing (equal). **Florian Lapalus:** Data curation (equal); investigation (equal); methodology (lead). **Didier Bouchon:** Formal analysis (supporting); investigation (supporting); methodology (supporting); software (supporting); validation (equal); writing – review and editing (supporting). **Géraldine Garcia:** Conceptualization (lead); data curation (equal); funding acquisition (equal); investigation (equal); methodology (equal); project administration (equal); supervision (lead); validation (equal); writing – review and editing (equal).

## CONFLICT OF INTEREST STATEMENT

The author declares no competing or financial interests.

## Data Availability

The supplementary tables, the raw datasets, and R code needed to reproduce all of the results reported in this paper are available from the Dryad Digital Repository (Gardin et al., 2023): https://doi.org/10.5061/dryad.9w0vt4bkw.
